# Desistance from Crime among Chinese Delinquents: The Integrated Effects of Family Bonding, Prosocial Models, and Religious Bonding

**DOI:** 10.3390/ijerph19105894

**Published:** 2022-05-12

**Authors:** Grace W. Y. Au, Dennis S. W. Wong

**Affiliations:** 1 School of Arts and Social Sciences, Hong Kong Metropolitan University, Hong Kong, China; 2 Department of Social and Behavioural Sciences, City University of Hong Kong, Hong Kong, China; dennis.wong@cityu.edu.hk

**Keywords:** crime and delinquency, crime prevention and intervention, offender rehabilitation

## Abstract

Desistance from crime is a popular topic in global criminological research; however, few studies have focused on desistance among delinquent youth, particularly in non-Western societies. This study extends the current knowledge by examining pathways of youth desistance in Hong Kong. Thirty delinquent youth and six parent–child dyads were interviewed, and the study found that filial piety significantly impacts the process of youth desistance. Three main forms of social capital were closely associated with youth desistance: the revival of reciprocal family bonding, the presence of a prosocial role model, and religious bonding. An interactive model was constructed to illustrate the seven stages of desistance and highlight the key elements for successful desistance among youth delinquents in Hong Kong.

## 1. Introduction

Desistance from crime and general offending has become an important topic of study in the criminological research over the past two decades, both theoretically and practically. Desistance from crime generally refers to the process of ending a period of involvement in offending behavior. Uncovering the underlying reasons for desistance from crime can contribute to reforming our current social control and crime prevention policies; in fact, several scholars believe that understanding desistance is as important as understanding the development of a criminal career itself [1,2,3]. Nevertheless, the academic literature on desistance is rare compared with that on criminal careers.

In studying the determinants of youth offending behavior, scholars [4,5,6,7] have found that social control variables, together with factors of differential association, are highly related to the emergence and continuation of criminal behavior. Sampson and Laub (2003) [8] asserted that significant changes in social control variables, such as the family, marriage, education, employment, peer relationships, community, and military services, may lead to the escalation or de-escalation of criminal careers. Desistance is a concept that focuses on how an individual manages to lead a crime-free life after a criminal career despite facing various obstacles [9]. Given that desistance from crime is not an easy goal to achieve in a singular attempt, this term commonly refers to the process of ending a period of offending behavior.

### 1.1. Factors Related to Successful Desistance

Among all the factors leading to successful desistance, familial and cultural values are seen as a crucial element in the process. Several studies have explored desistance processes among different ethnic or cultural groups, albeit mostly those living in Western countries. For instance, family is found to be a significant source of informal support that may promote desistance [10,11,12]. Cid and Marti (2012) [13] asserted that family plays a prominent role in the process of desistance, and family bonding could be a turning point by providing practical and emotional support, as well as motivating delinquents to stay away from crime in Spain. Based on in-depth interviews with 11 male Bangladeshi ex-prisoners in Tower Hamlets, England, Calverley (2013) [10] also discovered that family support was an important factor in youth desistance journeys.

In Hong Kong, scholars [14,15] have endeavored to explore factors that lead to the onset and continuation of juvenile delinquency, based on a theoretical framework of social process. Nevertheless, few studies have specifically examined whether integrated theories and religiosity can facilitate delinquent youths’ desistance from crime in Hong Kong. The knowledge that has been accumulated thus far seems to lack an understanding of how delinquent youth experience the desistance process and what barriers they may encounter when they are trying to abstain from crime. Recently, Adorjan and Chui (2012) [16] offered a preliminary empirical analysis of desistance from crime amongst prisoners in Hong Kong. They discovered that religiosity plays a significant role in helping ex-prisoners desist from crime. However, the study did not identify the factors contributing to youth desistance in Chinese societies. Similarly, Chui and Cheng (2014) [17] interviewed 16 young Chinese males within one year of their release from incarceration and identified the challenges they faced during community re-entry as a lack of family and other social supports. The significant role of parents is an important factor in desistance among delinquents as parents can provide different kinds of support to young people, and the family seems to be a key form of social capital in this process. Thus, the family is believed to be an essential source of informal support in Chinese societies that may sustain ex-offenders’ commitment to a crime-free life [10,11,12].

In particular, Chinese culture emphasizes family values [18], and Chinese families are distinct from those in Western culture for their adoption of filial piety (*xiao*) [19]. Under the influence of Confucianism, *xiao* refers to a set of behavioural prescriptions, such as showing respect, being obedient, honoring one’s parents and ancestors, taking care of one’s parents, and avoiding injuring oneself since one’s body belongs to one’s parents [20,21]. Thus, *xiao* is usually perceived as the most crucial ethical principle which everyone must value and practice. If the family is seen as one of the social control variables or forms of social capital that may encourage young people in Western cultures not to engage in crime, then the concept of *xiao* might also be a significant factor of successful desistance in Chinese societies. 

### 1.2. The Significance of Social Capital for Desistance

Equally importantly, the concept of social capital is seen as a significant element for enhancing young people’s social networks, which are formed in their neighborhoods, schools, and interest groups. Social capital is also believed to play an important role in enabling young people to overcome obstacles and move their lives in positive directions [22,23,24]. It has been noted that social capital is best operationalized as social connections among different kinds of significant others [25] or as a resource that emerges from people’s social ties [26]. Nonetheless, social capital has been adopted as an exogenous or causal research variable to differentiate its sustaining effects on desistance at different levels.

Bottoms and Shapland (2011) [12] suggested that desistance is influenced by individuals’ dispositions and their changing social capital (bonding and bridging), which can manifest as an opportunity to change. The authors asserted that reinforcing factors, which possibly emerge from within the individual or their social relationships, might form what Giordano et al. (2002) [2] defined as ‘hooks for change’ in the steps towards desistance. In the Sheffield desistance study, Bottoms and Shapland (2011) [12] confirmed that new social bonds and the strengthening of existing bonds appeared to be linked to successful desistance. The fourth and final stages of desistance, defined by Giordano et al., (2002: 1002) [2] entails ‘a transformation in the way the actor views the deviant behavior or lifestyle itself’. Similarly, in Vaughan’s (2007) [27] final stage of desistance, that of ‘dedication’, he argued that an individual must commit to their new identity; otherwise, desistance will not continue. According to Bottoms and Shapland’s (2011) [12] model, the fifth stage of desistance is subject to obstacles such as temptation that may lead to a relapse into offending, considering that renouncing crime is a very difficult process. Offenders’ social disadvantages under conditions such as poverty, health issues, or poor education may negatively affect the desistance process; therefore, Bottoms and Shapland (2011) [12] reiterated the importance of the need for reinforcing factors to strengthen individuals’ identity shifts during the final stage of this process.

### 1.3. Interactive Effects among the External and Internal Factors of Desistance

The process of desistance is now believed to be dependent on the interaction between external influences and an individual’s internal influences. External influences such as social and cultural factors are generally considered to be strong predictors that can either encourage or discourage the desistance process [28,29]. Meanwhile, evidence of individual identity changes and cognitive transformations is considered an important factor in internal influences [2,3]. Research has also found that religious bonding plays a significant role in helping ex-prisoners desist from criminal careers [30,31]. Taken together, external factors, individual identity changes, and religious bonding should not be neglected in the study of desistance. 

Though scholars have identified various important factors related to youth desistance from crime, it is unknown whether subjective factors such as ‘identity changes’ [2,3,32] or ‘agentic moves’ could be significant elements contributing to the construction of various turning points that encourage youth to desist [8]. In other words, the sequence of the appearance of different factors during the desistance process may be as important as the static factors themselves [33]. It is also believed that changes in both subjective and social factors may be reciprocal in terms of mutual interaction [3,34]; therefore, research on desistance has moved towards explaining desistance in terms of the interaction between internal and external factors. Recently, several studies on desistance models have been conducted in the United Kingdom. This strand of research has been growing slowly but steadily since 2001, grounded in a number of pioneering works such as Farrall’s (2002) [35] study on probationers, Maruna’s (2001) [3] Liverpool desistance study, LeBel et al.’s (2008) [32] research on male property offenders, and the Sheffield desistance study [12]. Conversely, the literature on desistance has often paid little attention to the desistance process of ethnic groups in non-Western societies. To fill the research gap, there is a need to identify an integrated theoretical model of desistance that is applicable to delinquent youth in the Chinese cultural context.

## 2. Methodology

The present study adopted a qualitative approach based on interviews with 30 male delinquent youth and six parent–delinquent dyads. Participants were recruited with the assistance of caseworkers from a non-governmental organization (NGO) which focuses on delinquency prevention and treatment in Hong Kong. The male youth in the six dyads had taken different paths to desistance, and such diversity was helpful in our efforts to understand desistance from a wider perspective. 

The study was approved by the Research Ethics Committee of the City University of Hong Kong. The interviews were conducted between January and October 2016, and based on an interview guide consisting of 30 semi-structured questions, they were mostly completed within 90 min. The key interview focus areas included the following: (1) How can youth offenders free themselves from crime and the transition from offending to desistance? (2) How did respondents utilize social support or social capital in their surroundings to achieve desistance? (3) What kinds of positive life histories and experiences did the respondents have when they encountered the NGO? Twenty interview questions were posed to each youth desister, most with a focus on their journeys towards desistance from crime. The one-on-one interviews with each respondent were conducted in a private room after informed consent was obtained. All interviews were conducted in Chinese and audio-recorded. The researchers translated the interviews verbatim into English.

In collecting data from the six parent–delinquent dyads, the parents and youth were interviewed separately, with two different sets of questions based on similar themes. The themes of sharing are mainly related to their own perceptions towards delinquency and desistance, parent–child communication patterns and behavioural changes before and after youth convictions. With the hope of collecting first-hand data from different sources to be coupled with the interviewees’ stories towards a multi-perspective understanding, such arrangements would allow the researchers to interpret the data more objectively. All interviews were conducted in Chinese and audio-recorded. The researchers translated the interviews verbatim into English.

Grounded theory [36] offered a strategic research direction for conducting the present study, and for formulating and refining the research framework systematically based on empirical data. Under the grounded theory-based methodological approach, data collection and data analysis usually run parallel and inform each other [37]. The preliminary framework of the present study includes individual offending histories, and external and internal factors of desistance such as family bonding and practices of filial piety, social connections with peer and significant others, religious belief, and agentic moves towards identity changes. With the framework in mind, the main purposes of this study are to understand the process of desistance from crime, find out factors of successful desistance, and identify an integrated model of desistance, among Chinese delinquents. Relevant research questions related to the framework of study are subsequently developed. At the end, the preliminary framework will be advanced based on integrated analysis of all results. 

As regards to procedures of data analysis, each transcript was read carefully, following the open code method [36]. Excerpts from each case that seemed relevant to the research questions were highlighted [38] and notes were also made in the margins of the transcripts. After an intensive first reading of some cases, researchers created a set of listening guides consisting of 6 major themes for cataloguing the rest of the research data. The six major themes include (1) social capital background, (2) criminal or deviant behavior history, (3) the journey of desistance from crime or deviant behavior, (4) obstacles to desistance from crime or deviant behavior, (5) perspectives on various factors of desistance from crime among young people, and (6) perspectives on the future.

## 3. Results

The ages of the 30 respondents ranged from 17 to 33 years, with a mean age of 22 years at the time of the interviews. All the young men who had previously manifested deviant behavior and offending were now either working or studying: they had stopped committing crimes or had not exhibited criminal behavior for at least one year, and there was a case in which one individual had desisted for 16 years (see Table 1). As shown in Table 2a,b, the respondents’ criminal/delinquent activities varied, including theft/robbery (33.3%), wounding/assault (23.3%), and trafficking in dangerous drugs (13.3%). Only three respondents were first-time offenders; their offences were sex crimes and criminal intimidation. Twenty-four respondents had previously been in custody, either on remand or due to sentencing. Four only had probation orders and had not been in custody before. Two were not convicted offending but had frequently run away from home and exhibited delinquent behaviors, such as assault or theft.

Regarding the respondents’ family backgrounds, 27 came from poor socio-economic backgrounds such as living in public housing flats or tiny cubicles. Sixteen came from traditional nuclear families, twelve lived in single-parent homes or had a step-parent, and two had been orphaned. Only one expressed that he had a good relationship with his parents, while 14 described their relationships with their parents as fair, and 13 said that they had poor or bad relationships with their parents. Two respondents were runaways, citing the feeling that their parents did not understand them as their reason for leaving home. Table 3 lists the basic demographic data of the six parent–delinquent dyads.

Three key factors were identified that seemed to impact the youth desistance process: a revival of reciprocal family bonding, the presence of a prosocial role model, and religious bonding. The present study’s findings revealed that there were different kinds of temptation, as well as ups and downs, during the desistance process; however, with family strong support, pro-social personnel, and Christian bonding, the desisters might overcome their challenges and remain on a morally correct path. 

### 3.1. The Revival of Reciprocal Family Bonding

The respondents identified family support as an important motivating factor for desistance. Twenty-four out of 30 respondents expressed that improved family relationships or having support from their caregivers were key factors that encouraged them to desist. Similarly, thirteen interviewees expressed that family support was an important factor that could enable them to develop strong motives to desist and subsequently commit to reforming their criminal identities. Hence, parents’ tears, unconditional love, and support represent major forces for change. Twenty-four out of 30 respondents said that when they were incarcerated in correctional institutions, they longed for visits from their family members; when their parents visited, they felt that they loved them and were concerned about their wellbeing. This unconditional love and support played an important role in motivating the young delinquents to transform themselves and attempt to become good sons. Some respondents also reported that they stopped their offending behavior to meet their obligations to their parents. Interestingly, this finding seems to suggest that the respondents attributed their desistance to the support they received from their families, and in return, they wanted to repay their parents by making positive changes, in line with a filial attitude. Y026, who had served a 160-h Community Service Order (CSO) for theft, also shared a similar view:

To fulfil the requirements of the CSO, I had to undertake unpaid work once per week while maintaining a six-day regular working schedule. During the Order period, I worked for my father’s company as a construction laborer. One day, we were working together on a project. I suddenly realized that my father was getting older and older, and he always looked exhausted while doing hard jobs. When I looked at his hair, it had all turned grey… [I realized] I needed to change my lifestyle immediately and reform myself as a responsible person.

Research has indicated that family support is a significant factor in the desistance process [39,40,41]. Nevertheless, when comparing the concept of ‘family support’ with that of ‘filial piety’, particularly in the Chinese context, filial piety seems to be more specific than the general concept of family support to describe parent–child bonding in Chinese communities. Under the influence of Confucianism, filial piety (*xiao*) refers to a set of behavioural prescriptions, such as showing respect, being obedient, honoring one’s parents and ancestors, taking care of one’s parents, and avoiding injuring oneself since one’s body belongs to one’s parents [20,21]. Yeh (2003) [42] proposed a dual filial piety model that identified two potentially opposing filial attributes, namely reciprocity and authoritarianism, in modern Chinese societies. Reciprocal filial piety (respect with love) encompasses the emotional and spiritual attendance to one’s parents out of gratitude and repayment for their sacrifices. On the other hand, authoritarian filial piety (respect with fear) is characterized by obedience, submission, and compliance, and encompasses financial and social responsibilities, as well as the obligation to continue the family bloodline and maintain a good reputation. As observed from the present research, incarceration led to improved family relationships, with some respondents mentioning that they became aware of their family’s concern for them during confinement. The boys greatly appreciated visits from their parents because this was the best way to keep in touch. Y021, who was imprisoned for robbery, said that his family was the strongest force behind his change in behavior:

When I was in the rehabilitation center, my parents and elder brother came to visit me nearly every week. During their visits, they felt sorry for my misbehavior and showed deep empathy and cried. I was touched and also felt ashamed, especially when my father, who is always tough in life, cried in front of me… I knew I was wrong and told myself that I could not give up and should turn over a new leaf.

The notion of reciprocity is a prevalent factor in filial piety, demonstrating that emotional bonding can bind parents and children together with mutual love and respect. This culture-specific value system is actively constructed and internalized through children’s experiences of parenting [43]. When asked why filial piety came to his mind, Y05, who was imprisoned for theft, shared:

In the good old days, I did not like my parents as they were harsh… After being incarcerated in a correctional institution, I recognized how good they actually are [and realized] I must change my attitudes towards them and show them reverence.

Chinese parents do not always express their love and emotions in words; on the contrary, they sometimes educate their children with strict attitudes. Consequently, miscommunication between parents and children can damage parent–child relationships. Twenty-seven out of 30 respondents expressed that they had poor relationships with their parents during their teenage years. Parenting that is perceived as strict and demanding is associated with authoritarian filial piety because it elicits only involuntary concessions to parental wishes [44,45]. However, when delinquents are imprisoned, their parents often express their love and show unconditional support for them to shed their criminal identities. These expressions of love and support trigger turning points that rejuvenate filial piety, positively affecting delinquents’ motivation to make changes in their lives. Twenty-four out of 30 described this as a revived reciprocal parent–child family bonding that supported their desistance aspirations.

### 3.2. Prosocial Role Models

Apart from a revival of family bonding, 21 out of 30 respondents revealed that they had a prosocial role model. These role models were professional helpers or life mentors who gave support for the respondents’ desistance. Bearing in mind that some of these young men came from single-parent families, they sometimes respected their prosocial role model more than their own parents. Y005 described how he met his prosocial role model and the positive influence he provided:

When I was detained, Michael, who is a worker from a religious organization, cared for me very much. He visited me nearly every week… When I was transferred to another correctional institution, he continued to visit me. I was empowered, [and I] felt supported and cared for. He encouraged me to start a new life after release, seeking a decent job, and not to reoffend.

The above example demonstrates that a prosocial role model can help delinquents going through a process of psychological restructuring and eventually help them build a positive self-concept and live a non-offending lifestyle. Several interviewees indicated that their relationships with their prosocial role models had encouraged them to think rationally and logically, in a way that was either new or, if not new, certainly not part of their normal repertoire. When asked how they thought they could make connections with positive peers, Y017, who was imprisoned for drug trafficking, explained:

Delinquents usually do not have positive peers because our former friends were not true friends… Nevertheless, I am sure that the friends we met in interest groups at the NGO were positive ones… I like to mingle with them and learn from them all the time.

21 out of 30 respondents met new friends and prosocial role models when they were serving their sentences or receiving counselling services. Having a prosocial role model, such as a life coach or positive acquaintance, seemed to enhance delinquents’ self-reflection. A prosocial role model sometimes also serves as a bridge for delinquents to rebuild their relationships with significant others. These interviewees shared that if there was adequate information and advice from helpers, they could take the opportunity to move forward. With the help of their prosocial role models, the majority of interviewees subsequently made changes to their delinquent lifestyles, and in return, their relationships with their parents were also improved. 

### 3.3. Religious Bonding

Religious support refers to the consideration, belonging, and spiritual and tangible support received from religious and faith-based organizations. Given the fact that Hong Kong was a British colony before returned to mainland China in 1997, Christianity has been playing a significant role in education and social welfare sectors for many years. According to statistics provided by Hong Kong Yearbook [46], the Protestant church community is currently running a total of 380 primary and secondary schools, accounting for 45% of the school population. Schools with a non-religious background account for around 40%, and the rest of schools are run by other religious bodies such as Buddhist and Taoist.

Fifteen of the interviewed young men became Christians during their desistance journey. They made at least some reference to Christian beliefs and were more likely to consider religious experiences as important catalysts for their change. Some said religious beliefs were a prominent theme in their experiences of desistance, while others ranked religion second or third in terms of the key factors associated with desistance. According to Campbell et al. (2007) [47], religious attachment not only has the potential to offer a solid conventional belief system and prosocial acquaintances but also provides a post-release institutional support network to help ex-offenders repair broken relationships, seek decent jobs, and find places to live, as well as offering basic social support. For instance, Y017 expressed his view of Christianity as follows:

After the first drug offence, I was sent to a treatment center for rehabilitation, but it did not help much. I also failed in my second attempt at rehabilitation. I committed another drug-related offence and was put under probation during my third attempt… The probation officer referred me to a Christian NGO for gospel treatment. I stayed there for more than a year and accepted Christ as the Lord of my life. Christian belief taught me right from wrong and rebuilt the relationship between me and my father.

The above quotation suggests that strengthening one’s commitment to religious faith could be linked to the reduction in delinquency and drug use, a finding that is in line with previous research [48,49]. Religious bonds may also facilitate successful community re-entry for ex-offenders [50,51]. Another example from Y014, a former triad gang member, suggested that religious bonding helped him avoid further associations with delinquent peers. He was invited to participate in different religious activities with the encouragement of a prosocial role model that he met during his imprisonment. After release, Y014 continued to attend church activities. Y018, who received a two-year probation order for theft, also claimed that his faith in Jesus was the major contributory factor to his successful desistance:

The school that I previously attended had a Christian background. One day I decided to play truant, and I went to a chapel for a short rest. The environment reminded me about my good old days in student fellowship. I sat in the chapel for a while; suddenly, I felt that I was filled with the Spirit… Tears were falling from my eyes, and I felt sorry for what I had done. I ran back to my class master and apologized for not studying hard. From that day, I stopped my bad behavior and became a devoted Christian.

The present study’s results show that religious bonding is another important factor during the desistance journey. Some youth delinquents shared that religious volunteers were extremely important in leading them to rebuild a new life, aided by all kinds of support and encouragement. Meanwhile, other interviewees also suggested that their religious faith helped them break free from undesirable peer relationships since their beliefs in God does not allow them to mingle with bad people or do immoral things. 

### 3.4. Difference in Interpretations of Delinquency Behaviour between Parents and Their Children

Analysis of the data collected from the six parent–delinquent dyads offered insights into the discrepant perceptions between parents and young people regarding the same delinquent behavior. The excerpts below suggest that their interpretations were interwoven when they reflected on the seriousness of the young people’s former delinquent activity. For example, Y001 lived with his father until the age of 19. From the first year of secondary school, he was addicted to online games and started playing truant and running away from home. He committed theft and was cautioned twice by a police superintendent in the following years. He was then put on probation and detained in the probation home due to theft and running away from the home twice. During the interview, Y001 described himself as follows:

I was not a good boy… I was crazy for online games [and committed] violent acts in my teenage years. When my father attempted to control my behavior, I reacted by breaking furniture at home and eventually ran away.

Conversely, the boy’s father (F029) did not have such a bad impression of his son:

Our relationship was relatively good. I treated him as a friend more than a son. Actually, he would listen to what I said, even when he was going through the stormy teenage stage.

A few examples from the interviews suggested that a certain degree of discrepancy in the perception of the youths’ delinquency was obvious. Y006 became a delinquent when he was in the second year of secondary school. He fell in with a group of triad gang members and began smoking, fighting, and using foul language. He was imprisoned for drug trafficking when he was 17 years old. He described himself as follows:

I was really bad when I was young. I sold drugs and was frequently involved in gang fighting. I would have liked to stop; unfortunately, I didn’t of my own accord but was eventually caught by the police.

His mother (F010) recalled her disbelief at the time and how she felt that her son was still a good person:

I was shocked when I was informed about my son’s troubles with the police. I did not accept the fact that my son had been involved in many different kinds of delinquency. He was not bad by nature, he just didn’t like studying.

The above excerpts suggested that the parents tended to protect their children’s image, trying very hard to avoid criticizing their son’s negative behaviors in front of others. This may be due to the passion that is concomitant with the reflection of a sense of parental concern. To a certain degree, the parents clung to the positive perceptions of their sons that manifest among parents when they are asked to comment on their sons’ behaviors in front of the authorities, glossing over the negatives. Underlying this, however, there may be another reason for the parents’ attempts: in Chinese society, having been socialized to respect the values of filial piety, individuals are obligated to protect their families against external threats. Filial piety demands obedience, honoring one’s parents, loving family members, and avoiding bringing disgrace to the family. As such, a single delinquent act is usually not attributed to the individual’s own nature, but rather to the external factor of the family environment. Should there be wrongdoings within the family circle, the entire family will lose ‘face’ when one member is heavily criticized and stigmatized by others for their criminal behavior [52]. A publicly shamed person can regain ‘face’ only by improving their reputation by clarifying the circumstances and doing good deeds in the family circle, hopefully earning back recognition from others [52,53]. 

### 3.5. The Integrated Strength of Parental Support, Filial Piety, and Religious Bonding

The key factor in the six young men’s desistance experiences was that their families (parents) were central to their reform. Parents’ forgiving attitudes towards their convicted children were critical in enabling the delinquents’ desistance. The parents reported that despite their negative feelings about their children’s delinquent behavior, they were able, with professional workers’ help, to change their attitudes and communication styles in ways that encouraged their children to consider opportunities for a crime-free future. Agentic willingness with respect to filial piety, which involved ‘hooks for change’ [2], was crucial for the delinquents as they moved through the desistance process, as was the increased awareness of having some degree of control over their lives after release; that is, they understood that if they took the right steps, they could ensure that this type of future became a reality. Their parents’ attitudes and the prisons/institutions in which they had been incarcerated fostered a sense of agency within them. This not only facilitated these young men’s initial desire to desist from crime but also generated a sense of personal gratitude among them towards their parents and positive acquaintances for their forgiveness, leading to further incentives for the desisters to maintain their efforts.

Kegan (1994) [54] argued that cognitive transformation goes beyond acquiring knowledge; instead, he asserted that the socialization that is reflected in one’s mind is linked to every aspect of a person’s life. The present study also confirmed that when desisters experience a certain degree of religious bonding, they can gain a clear set of norms to follow and are more likely to sustain their motivation to desist from crime. The process of acquiring religious beliefs seems to be somewhat equivalent to a kind of cognitive transformation, in which shared rituals and beliefs adhere to the individual [55,56]. Connecting regularly with churches or other religious bodies may encourage the receipt of support and a sense of connectedness through ties with other congregants [57], thereby minimizing vulnerable persons’ opportunities to routinely engage in deviant conduct [48,58].

In addition, with purposeful interventions by professional helpers, such as parental counselling and religious programs run by the Christian NGO, the young delinquents were empowered and able to overcome stigmatization. The NGO’s interventions added value to reinforce positive influences that assisted the delinquents, and their family members ultimately rebuilt their self-images and regained hope for the future. A beam of light shone on the affected families, imbuing them with aspirations for positive changes in the delinquents’ life trajectories. Revived family bonding, through trust bonds and improved parental relationships, granted the desisters access to social capitals that they could draw upon to maintain their desistance. These social capitals included emotional support, such as offering encouragement and certifying their achievements, practical support such as help with finances and accommodation, and the offering of structured roles to fill, such as becoming a good or filial son. This powerful effect results from experiencing parents’ love and filial piety. With positive parental care, desisters became able to think and reflect deeply (hooks for change) and make their own decisions (willingness to change).

### 3.6. A Model of Desistance Stages for Chinese Delinquents

**Figure 1:**
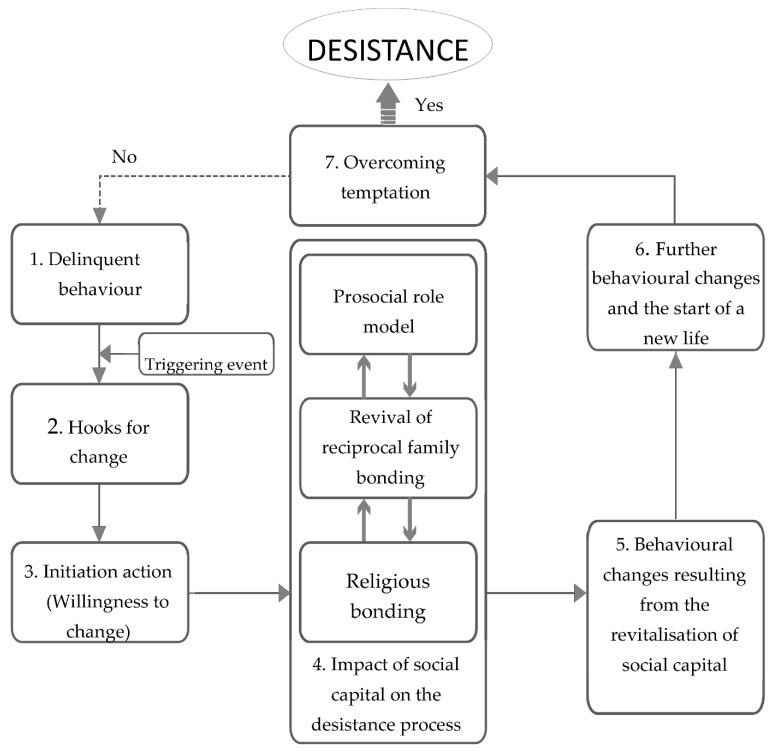
A Model of Stages of Desistance among Hong Kong Youth.

Since the beginning of the 2000s, scholars have endeavored to construct models of desistance from crime that incorporate the characteristics and challenges of its different stages [2,12,27]. Based on the present study’s findings, the researcher incorporated some innovative elements that are relevant to the Chinese cultural context into the existing models of desistance. Using ideas borrowed from the Sheffield desistance study [12], Figure 1 presents a model that illustrates the seven-stage desistance process among youth delinquents in Hong Kong.

Figure 1 clearly shows desisters’ pathways across different stages of the desistance process. The journey begins with an individual having been either in custody or detained due to delinquency (Stage 1). After the triggering event, the youth delinquent develops ‘hooks for change’ and takes action to affect actual changes. The three major factors of desistance are seen at the center of the diagram: (a) a revival of reciprocal family bonding, (b) prosocial role models, and (c) religious bonding. The interactional effects of these factors seem to create a positive desistance process, which can also be seen as a process of cognitive transformation. This finding is consistent with previous findings on youth desistance, suggesting that with tangible and moral support from various sources of social capital provided, a successful cognitive transformation may further bolster desisters’ efforts to adopt prosocial, crime-free lifestyles [12,59,60].

Similar to Giordano et al.’s observation (2002) [2], the initial steps in Stages 2 and 3 seem to be more cognitive; for example, the inner power of filial piety was also found to exert its impact from Stage 4 onwards. There may be repercussions in the progress of desistance: if a relapse occurs, the desister returns to their starting point. However, when an individual’s bonding is strengthened (Stage 4), their new identity can be generated and other subsequent positive attitudinal and/or behavioural changes follow (Stages 5 and 6). At Stage 7, desisters’ goals and obligations might help them overcome temptation and stand firm in their continued desistance from crime. Given that the present research was a qualitative study based on an interpretivist approach and using a small sample, it was not possible to identify the causal mechanism of youth desistance. However, the results clearly showed that most desisters underwent cognitive change in terms of desisting from offending before they achieved behavioural change. This result offers insight into the sequence of the desistance process through an understanding of which factors precede desistance itself.

## 4. Discussions

Filial piety was found to be an invisible force that substantially impacted the desistance process. In the Chinese cultural context, the family remains the main source of social control with regard to child development and assumes primary responsibility for socializing children [61]. According to Chen (2014) [62], children who perceive their parents as warm and rational are more likely to develop a reciprocal, affectionate relationship with them (reciprocal filial piety); nevertheless, they also recognize that as a son or daughter, they have a family duty to show their parents respect (authoritarian filial piety). Under the influence of Confucianism, crime is not seen as an individual problem; rather, the causes of crime are strongly related to the entire family’s parenting, bonding, and familial culture, instead of being confined to the offender. Therefore, when it comes to the resolution of crimes, entire families, rather than just individual perpetrators, are held responsible. 

It was also noted that the presence of a prosocial role model could contribute to encouraging delinquents to maintain hope for the future and sustain their willingness to stop committing delinquent acts. This finding concurs with that of Farrall and Bowling (1999) [63]—that the development of positive, supportive relationships during incarceration could help ex-offenders relinquish their deviant lifestyles. Interestingly, Barry (2013) [64] mentioned that the desistance process is becoming individualized, that is, young desisters are expected to stop offending of their own accord after undertaking reflection on their lives. In the present study, we also found a similar situation in which some desisters made positive agentic moves on their own, including seeking help to rebuild their relationships with their parents and maintain their supportive relationships after certain life encounters.

In addition, social capital, including individuals’ social ties and connections, is essential. The present study found that religious bonds could provide ex-offenders with emotional and psychological support, and religious activities can serve as a rehabilitation pillar in the desistance process [65,66]. The results also revealed different kinds of temptations during the desistance process; nevertheless, with strong support from the family, pro-social personnel, and religious organizations, desisters were able to overcome such challenges and remain on the right track towards a healthy lifestyle. These results align with Maruna’s (2001) [3] observations that when delinquents perceive strong, positive support from significant people in their lives, they are better equipped to develop and utilize ‘hooks for change’ during early-stage desistance [2]. Similarly, the finding concurs with LeBel et al.’s (2008) [32] idea that desisters’ hopefulness, together with a supportive agentic role in the process of identity reconstruction, constitute key elements of desistance. 

In the past two decades, scholarly studies on desistance from crime have gained much attention; nevertheless, a solid theoretical foundation across different cultural contexts has remained underdeveloped [67,68,69]. To fill this research gap, the present study undertook a pioneer examination of key elements of social capital that are pertinent to Chinese delinquents’ desistance processes. Such findings may provide important references for other Asian countries, such as Mainland China, Singapore, Taiwan, Japan, and Korea, contributing to the overall advancement of the desistance theory. Most importantly, the study’s findings have confirmed the significant roles played by family bonding, prosocial modelling, and religious bonding in the desistance process. The model confirmed our previous theoretical conceptions that youth delinquents’ families are the most influential agents of change and may affect the continuation or desistance of a delinquent’s criminal career. Hence, helping young delinquents maintain or restore positive relationships with their family members should be a central point of professional interventions aimed at youth desistance from crime. 

The limitations of this study include mainly relying on retrospective data collection, the veracity of which is dependent on the accuracy of respondents’ memories regarding their life experiences, and the accuracy of these recollections may have been compromised. Due to the sensitive nature of the study, purposive sampling was adopted in the present research instead of probability sampling. The findings are not easily generalizable to a wider population. Furthermore, employment/education was also not deeply explored in the present study. Laub and Sampson (2003) [8] have suggested that employment characteristics, such as commitment to work, ties with employers, and relationships with colleagues, are all important in the process of desisting from crime. Thus, future research should not exclude this area of study. Finally, it pertains to gender because the youth desister respondents were all male, and the majority of the key family members were female, i.e., mothers. It would be insightful for future researchers to examine the similarities and differences in the male versus female desistance process in terms of the challenges and barriers that delinquents encounter.

## 5. Conclusions

The role of social capital was found to intersect with delinquents’ life trajectories by helping to create turning points in their individual desistance processes. The revival of social networks was not only important in terms of providing emotional support—it was also essential in giving an individual more opportunity and choices, and ultimately, the power to desist. The present study confirmed that desistance from crime is not just the permanent cessation of criminal activity; rather, it is a process of acquiring virtues and new social identities and developing new social ties. Consistent with previous literature, the present study discovered that family support plays a significant role in youth desistance. There are three main forms of social capital that are closely associated with youth desistance in Chinese society: the revival of reciprocal family bonding, the presence of a prosocial role model, and religious bonding. Revived reciprocal family bonding is linked to the concept of reciprocal filial piety, which is the most influential form of social capital, compared to the study’s other research variables. It helps delinquent youth free themselves from the vicious cycle of negative labelling, thus enabling desisters to reclaim positive statuses that are far removed from their illicit pasts.

The present study concluded that the presence of appropriate social capital seems to have the power to bridge and link desisters’ social bonds. The majority of delinquent youth appear to have poor parent–child attachments or dysfunctional familial relationships. This study’s findings suggested that social capital plays a key role in effectively bridging the gap between parents and delinquents, so that desisted youth can restore their relationships with significant others in society. This can certainly help boost a revival of their reciprocal family bonds, and in turn, a revival of other social bonds. Reciprocal filial piety is found to have a beneficial impact on motivating desisters to liberate themselves from criminal lifestyles. Furthermore, the present study also found that a prosocial role model for desistance and religious bonding could consolidate an individual’s commitment to desistance and to themselves becoming a social capital source that can redirect other delinquent youth into a more conventional life. Based on the current findings, the researchers constructed an interactive model of different stages of desistance that illustrates Chinese youth desistance characteristics. The model depicts seven stages of desistance, reflecting the desistance process that is operational among youth delinquents in Hong Kong.

## Figures and Tables

**Table 1:** Delinquent Youth Sample Characteristics.

Code	Age	Offence	Desistance from Crime (Year)	Employed or Attending School
Y001	20	Theft	2	Yes
Y002	19	Theft (shop theft)	7	Yes
Y003	19	Assault	2	Yes
Y004	21	Claiming to be a triad member	5	Yes
Y005	26	Theft	8	Yes
Y006	22	Trafficking in dangerous drugs	4	Yes
Y007	20	Theft	5	Yes
Y008	17	Assault	3	Yes
Y009	20	Criminal intimidation	4	Yes
Y010	18	Assault	3	Yes
Y011	27	Indecent assault	4	Yes
Y012	24	Theft (employee embezzlement)	3	Yes
Y013	23	Runaway from home * (and assault)	6	Yes
Y014	25	Claiming to be a triad member	3.5	Yes
Y015	21	Assault	3	Yes
Y016	26	Assault	5	Yes
Y017	23	Trafficking in dangerous drugs	1.5	Yes
Y018	19	Taking conveyance without authority	1.5	Yes
Y019	21	Fighting	7	Yes
Y020	19	Indecent assault	4	Yes
Y021	24	Robbery	1.5	Yes
Y022	19	Criminal intimidation	1	Yes
Y023	18	Runaway from home * (and theft)	1	Yes
Y024	21	Trafficking in dangerous drugs	4	Yes
Y025	26	Other miscellaneous theft	10	Yes
Y026	33	Other miscellaneous theft	12	Yes
Y027	22	Possession of an offensive weapon and assault	2.5	Yes
Y028	23	Assault	6	Yes
Y029	31	Theft	16	Yes
Y030	17	Trafficking in dangerous drugs	1.5	Yes

* These two persons were under Protection Order before their desistance as they had been running away from home for a few times. Based on the welfare practices in Hong Kong, frequent running away acts among adolescents would be considered as delinquent behavior. The Order was granted by the court for their needs of care or protection, based on the Protection of Children and Juveniles Ordinance (Cap. 213).

**Table 2:** (**a**) Demographic characteristics at the time of interview (*n* = 30); (**b**) Male Respondents’ Demographic Characteristics (*n* = 30).

(**a**)		
**Variables**	**Number of Young Men**	**%**
*Age*		
11–20	12	40
21–30	16	53.3
31–40	2	6.7
	*n* = 30	100
*Offence/delinquent act*		
Theft/robbery	10	33.3
Assault	7	23.3
Trafficking in dangerous drugs	4	13.3
Criminal intimidation	2	6.7
Indecent assault	2	6.7
Running away from home (and others)	2	6.7
Claiming to be a triad member	2	6.7
Fighting	1	3.3
	*n* = 30	100
*Family background*		
Living with both parents	16	53.3
Living with a single parent	10	33.3
Living with a stepparent	2	6.7
Orphaned	2	6.7
	*n* = 30	100
*Relationship with parents*		
Good	1	3.3
Fair	14	46.7
Poor	8	26.7
Very Bad	5	16.7
None	2	6.7
	*n* = 30	100
(**b**)		
**Variables**	**Number of Young Men**	**%**
*Last sentence or referral at time of interview*		
Boys’ home (SWD)	6	20
Probation order/community service order (SWD)	5	16.7
Probation home (SWD)	1	3.3
Social development school	3	10
Correctional institution (CSD)	6	20
Detention center (CSD)	2	6.7
Training center (CSD)	3	10
Drug addiction treatment center (CSD)	2	6.7
Rehabilitation center (CSD)	2	6.7
	*n* = 30	100
*Desistance from crime (years)*		
1–5	22	73.3
6–10	6	20
11–20	2	6.7
	*n* = 30	100
*Embraced bonding to religious belief after sentencing or referral*		
Yes	15	50
No	15	50
	*n* = 30	100

**Table 3:** Parent–Child Dyads’ Demographics.

Code	Offence/Delinquent Act for Last Sentence or Referral	Desistance (Year)	Desister Became a Christian	Code	Parent	Parent Became a Christian
Y001	Theft	2	Yes	F029	Father	No
Y005	Theft	8	No	F030	Mother	No
Y006	Trafficking in dangerous drugs	4	Yes	F010	Mother	Yes
Y008	Assault	3	No	F015	Mother	Yes
Y010	Assault	3	No	F019	Mother	Yes
Y020	Indecent assault	4	No	F005	Mother	Yes

## Data Availability

The data presented in this study are available on request from the corresponding author. The data are not publicly available due to participant privacy.
